# Bildung von erkrankungs- und bedarfsbezogen homogenen Versichertengruppen – PopGrouper 1.0

**DOI:** 10.1055/a-2872-0161

**Published:** 2026-06-19

**Authors:** Anna Braun, Thomas G. Grobe, Chrissa Tsatsaronis, Peter Reschke, Karen Kinder, Maria Klemt, Danny Wende, Claudia Schulte, Klaus Focke, Edgar Steiger, Mandy Schulz, Thomas Czihal, Wilm Quentin, Reinhard Busse

**Affiliations:** 1 Abt. Gesundheitsberichterstattung und Biometrie, aQua – Institut für angewandte Qualitätsförderung und Forschung im Gesundheitswesen, Göttingen, Germany; 2 Fachgebiet Management im Gesundheitswesen, Technische Universität Berlin, Berlin, Germany; 3 Gesundheitssystemforschung, BARMER Institut für Gesundheitssystemforschung, Dresden, Germany; 4 Gesundheitssystemforschung, BARMER Institut für Gesundheitssystemforschung, Berlin, Germany; 5 Data Science, Zentralinstitut für die kassenärztliche Versorgung, Berlin, Germany; 6 Versorgungsanalysen, Zentralinstitut fur die kassenarztliche Versorgung in Deutschland, Berlin, Germany; 7 Versorgungsforschung und Risikostruktur, Zentralinstitut für die kassenärztliche Versorgung, Berlin, Germany

**Keywords:** Bevölkerungsgruppierung, Morbidität, Gesundheitskosten, Entscheidungslisten, Regressionsbäume, Routinedaten, Population grouping, morbidity, healthcare costs, decision lists, regression trees, routine data

## Abstract

**Ziel:**

Für eine differenzierte Erhebung und Darstellung des Versorgungsbedarfs werden in verschiedenen Ländern bevölkerungsbezogene Klassifikationssysteme genutzt. Das PopGroup Projekt hatte zum Ziel, ein solches Klassifikationssystem für den deutschen Versorgungskontext zu entwickeln. Der PopGrouper liegt zwischenzeitlich in der Version 1.0 vor und ordnet jede Person genau einer PopGroup (PG) zu. Nach einer vorausgehenden Publikation zu Gruppierungen von Erkrankungen mit Zuordnungen von Schweregraden sollen im vorliegenden Artikel Folgeschritte zur exklusiven Gruppierung aller Versicherten auf mehreren Ebenen vorgestellt und erläutert werden, womit die methodische Beschreibung des Groupers komplettiert wird.

**Methodik:**

Für die Entwicklung der Version 1.0 wurden Trainings- und Testdaten zu insgesamt 8,8 Mio. Versicherten der BARMER-Krankenkasse im Verhältnis 4 zu 1 aus dem Jahr 2022 genutzt. Nach einer inhaltlich-klinisch orientierten Gruppierung erfolgten weitere Aufteilungen algorithmisch, wozu in Trainingsdaten mehrfach sog. Entscheidungslisten und Regressionsbäume mit spezifischer Implementierung genutzt wurden. So konnte ein Regelwerk erstellt werden, das auch zur Gruppierung anderweitiger Populationen geeignet ist.

**Ergebnisse:**

Alle Personen wurden auf 4 hierarchischen Ebenen jeweils genau einer von 10, 427, bzw. 610 Gruppen sowie abschließend genau einer der 776 PGs zugeordnet, die in Testdaten 38,8% der Varianz von versichertenbezogen dokumentierten Kosten für die gesundheitliche Versorgung erklärten. Nach Anwendung der Gruppierungsregeln in Daten zu 2021 sowie 2023 zeigten sich ähnliche Ergebnisse, wobei erklärte Varianzanteile auf allen Gruppierungsebenen in Daten zu 2023 tendenziell höher als 2022 lagen.

**Schlussfolgerung:**

Mit dem PopGrouper 1.0 liegt erstmals ein für Daten der gesetzlichen Krankenversicherung (GKV) in Deutschland angepasster bevölkerungsbezogener Grouper vor, der umfassend dokumentiert ist und frei genutzt werden kann. Ergebnisse deuten auf gut nutzbare Gruppierungen für die Datenjahre 2021 bis 2023 hin, für die bereits vollständige Gruppierungsregeln existieren. Anpassungen für Folgejahre sollten angestrebt werden, wobei geprüft werden könnte, inwiefern Vereinfachungen des Groupers und seiner Pflege ohne Qualitätsverluste möglich sind.

## Abstract (en)

**Objective:**

Population-based classification systems are used in various countries to assess and present healthcare needs. The PopGroup project aimed to develop such a classification system for the German healthcare context. Version 1.0 of the PopGrouper is now available and assigns each person to exactly one PopGroup (PG). This article builds on a previous publication on the grouping of diseases with assignments of severity levels and explains the next steps for the mutually exclusive grouping of all insured persons across several levels, thus completing the methodological description of the grouper.

**Methodology:**

For the development of version 1.0, training and test data from a total of 8.8 million insured persons of the BARMER health insurance fund for the year 2022 were used in a ratio of 4 to 1. After an initial content and clinically oriented grouping, further splits were carried out algorithmically using decision lists and regression trees. This resulted in a set of rules that can be used also for grouping of other populations.

**Results:**

All individuals were assigned to exactly one of 10, 427, or 610 groups and finally into 776 PGs across 4 hierarchical levels. PGs explained 38.8% of the variance in insurance-related documented health care costs in test data. After applying the grouping rules to data from 2021 and 2023, the results were similar, with explained variance shares across all grouping levels tending to be higher in 2023 than in 2022.

**Conclusion:**

The PopGrouper 1.0 is the first population-based grouper adapted for statutory health insurance data in Germany that is comprehensively documented and free to use. The grouper has proven useful for data from the years 2021 to 2023, for which complete grouping rules already exist. The grouper should be updated for future years, while also examining to what extent simplifications of the grouper and its maintenance are possible without loss of quality.

## Einleitung

Zur Ermittlung gesundheitlicher Versorgungsbedarfe und für die Versorgungsplanung werden international häufig bevölkerungsbezogene Grouper verwendet, welche Populationen – typischerweise mit Rückgriff auf Informationen aus Krankenversicherungssystemen – in diskrete Gruppen teilen 1 2 3 . Entsprechende Zellenmodelle mit Zuordnungen aller Versicherten zu jeweils genau einer Gruppe (bzw. Zelle) bieten gegenüber Zuschlagsmodellen wie dem morbiditätsorientierten Risikostrukturausgleich (Morbi-RSA; 4 ) i.d.R. den Vorteil, dass auch ein Zusammenwirken unterschiedlicher Merkmale abgebildet wird, da in den Zellen stets Personen mit all ihren Eigenschaften betrachtet werden. Zudem können für die Gruppen auch im Nachhinein noch spezifische Bedarfe ermittelt werden, aus denen sich dann erwartete Bedarfe auch für solche Leistungen ableiten lassen, die bei der eigentlichen Entwicklung des Groupers nicht berücksichtigt wurden (z. B. Bedarfe für bestimmte stationäre Versorgungsangebote). Ähnlich wie ein Matching ermöglicht ein einmal entwickeltes Zellenmodell damit eine Vielzahl an Gegenüberstellungen, wohingegen regressionsanalytische Ansätze bei Berücksichtigung neuer Merkmale stets Neuberechnungen der Modelle erfordern. Das vom Innovationsfonds geförderte PopGroup Projekt hatte zum Ziel, ein solches bevölkerungsbezogenes Klassifikationssystem (den PopGrouper) für den deutschen Versorgungskontext zu entwickeln 5 . Dieser liegt zwischenzeitlich in der Version 1.0 vor und ordnet jeden Versicherten basierend auf Daten zu einem Kalenderjahr auf der am weitesten differenzierten Ebene genau einer von maximal 776 disjunkten Gruppen – den sogenannten PopGroups (PGs) – zu.

Die Entwicklung des PopGroupers lässt sich zwei grundlegenden Entwicklungs- bzw. Arbeitsschritten zuordnen, die gleichermaßen auch bei der Nachbildung, und damit bei einer Verwendung von PGs mit neuen Versichertendaten, durchlaufen werden müssen:

- In einem ersten Arbeitsschritt werden in Daten verfügbare Diagnoseangaben maßgeblich nach fachlich-medizinisch sinnvollen Kriterien gruppiert, wobei einzelnen Versicherten ggf. auch eine größere Zahl an Diagnosegruppen zugeordnet sein kann.
- Im zweiten Arbeitsschritt erfolgt – maßgeblich basierend auf Zuordnungen von Diagnosegruppen aus Arbeitsschritt I – die Zuordnung der Versicherten zu disjunkten Gruppen auf insgesamt vier hierarchischen Ebenen, wobei die am stärksten ausdifferenzierte Ebene dann von PGs gebildet wird.

Arbeitsschritt I mit Bildung von 433 sogenannten Zusammengefassten Krankheitsgruppen (ZKGs) dient dazu, eine handhabbare Anzahl von „Morbiditätsbausteinen“ in medizinisch sinnvollen Gruppen zu schaffen. Den ZKGs wird dabei jeweils auch einer von 5 Schweregraden (von sehr leicht über moderat bis sehr schwer) sowie eine von insgesamt 32 Makrokrankheitsgruppen (MKG) zugeordnet. Letztere bilden eine übergeordnete Gliederungsebene in Anlehnung an Facharztdisziplinen bzw. medizinische Versorgungsbereiche. Das Vorgehen bis zur ZKG-Bildung ist ausführlich im Beitrag von Tsatsaronis et al. im Gesundheitswesen mit dem Titel „Definition zusammengefasster Krankheitsgruppen für ein Klassifikationssystem zur Messung des morbiditätsbezogenen Versorgungsbedarfs – PopGroup“ dargestellt 6 .

### Exklusive Gruppierung von Versicherten

Der vorliegende Beitrag befasst sich demgegenüber mit Arbeitsschritt II, nämlich der Zuordnung jedes Versicherten zu jeweils genau einer Gruppe auf insgesamt vier hierarchischen Gruppierungsebenen sowie zu einer ergänzenden, kostenbezogenen Gruppierung von PGs. Mit dem Beitrag wird insofern die methodische Beschreibung des PopGroupers komplettiert. Mit der Zuordnung der Versicherten zu PGs wurden folgende Ziele angestrebt.

Jede PG soll im Hinblick auf das Erkrankungsspektrum sowie die gesundheitlichen Versorgungsbedarfe möglichst homogene Gruppen von Versicherten umfassen.
Jede PG soll eine relevante Anzahl an Versicherten enthalten und absehbar möglichst auch über längere Zeiträume als Gruppierung Bestand haben.
Jeder Versicherte soll innerhalb eines Kalenderjahres genau einer PG zugeordnet sein, was – wie beim Morbi-RSA – auch für unterjährig Versicherte gelten soll.
Jede PG soll inhaltlich-medizinisch möglichst sinnhaft, nachvollziehbar und intuitiv verständlich gebildet und benannt sein.
Bei PGs sollen explizit relevante Erkrankungskombinationen berücksichtigt werden.
Merkmale, die außerhalb von GKV-Daten kaum verfügbar sind, sollen ausschließlich zur Differenzierung von PGs herangezogen werden, womit übergeordnete Gruppierungsebenen auch ohne derartige Daten nachgebildet werden können.

Das Ziel des vorliegenden Beitrags ist es, die bei der Entwicklung des PopGroupers gewählten Vorgehensweisen verständlich zu machen und Einblicke in den Aufbau des Groupers und dessen Eigenschaften zu vermitteln. Dabei können zwangsläufig nicht alle im Rahmen der Entwicklung abgewogenen und teils verworfenen Ansätze erläutert werden. Die Darstellung beschränkt sich i.d.R. auf Erläuterungen zur finalen Version 1.0 und zu dabei verwendeten Daten. Aus Sicht der Beteiligten war die Entwicklung des PopGroupers in jedem Fall ein Forschungsprojekt im besten Sinne, bei dem das Resultat zu Beginn im Detail kaum absehbar war und ohne den Input eines engagierten Teams nicht möglich gewesen wäre.

## Methodik

### Verwendete Daten

Zur Entwicklung des PopGroupers 1.0 wurden pseudonymisierte und datenschutzkonform abgelegte Daten im Wissenschafts-Data Warehouse (W-DWH) der BARMER zum Beobachtungsjahr 2022 genutzt. Alle Datenaufbereitungen und -analysen wurden in SAS-Software in der Version 9.4 durchgeführt. Eingeschlossen wurden insgesamt n=8 838 141 inländisch wohnhafte und mindestens einen Tag Versicherte mit konsistenten Geschlechtsangaben im Alter von 0 bis<125 Jahren (Alter=2022 – Geburtsjahr). Zur Entwicklung des Groupers wurde eine Zufallsauswahl von Daten zu 80% der Versicherten verwendet (n=7 070 986; Trainingsdaten, TrD); Daten zu 20% wurden ausschließlich als Testdaten verwendet (TeD, n=1 767 155). Versicherte in Trainingsdaten waren zu 56,5% Frauen (TeD: 56,4%); das Durchschnittsalter lag bei 48,2 Jahren (TeD: 48,2). Im Mittel waren Personen 353,3 Tage (TeD: 353,4) und damit 96,5% aller Tage (TeD: 96,5%) innerhalb des Jahres bei der BARMER versichert. Zur Bildung von PopGroups wurde auf die Merkmale Geschlecht, Alter und diagnosebezogene Zuordnungen aus Arbeitsschritt I (ZKG, MKG – inklusive von Angaben zu Schweregraden) sowie Angaben zu Gesamtkosten zurückgegriffen. Ausschließlich zur finalen Ausdifferenzierung von PopGroups wurden, im Einklang mit Ziel Z6, zudem die in Tab. 1 gelisteten Merkmale verwendet.

**Table:** **Tab. 1** Merkmale zur finalen PopGroup-Differenzierung – Anteil Betroffene 2022.

Merkmal [Einheit, Kodierung]	Anteil betroffen	Erläuterung
Pflegegrad [ordinal]	8,85% (8,82%)	Einteilung in sechs Pflegegrade von 0 bis 5 gem. SGB XI, jeweils der zuletzt beobachtete Pflegegrad im Beobachtungsjahr (Basiswert=0; entspricht Fehlen eines Pflegegrads).
Beatmung [Stunden, gruppiert]	0,32% (0,32%)	Beatmungsstunden bei Behandlungen in Krankenhäusern, Daten gem. § 301 SGB V, Angaben in 10 Gruppen mit folgenden Obergrenzen: 0, 24, 48, 72, 95, 249, 499, 999, 1799, ab 1800 (Basiswert=0)
Ind_Komplex [0/1]	0,69% (0,69%)	Komplexbehandlung in Krankenhäusern, 0/1-kodierte Indikatorvariable, 1 falls folgende OPS-Kodes in Daten gem. § 301 SGB V: 8-980, 8-98 d, 8-98 f (Basiswert=0)
Ind_Insulin [0/1]	2,54% (2,55%)	Insulinbehandlung, 0/1-kodierte Indikatorvariable, 1 falls folgende ATC-Kodes in Daten zu Arzneiverordnungen aus Apotheken gem. § 300 SGB V: A10AB, A10AC, A10AD, A10AE, A10AF
Ind_Metabol [0/1]	0,39% (0,40%)	Behandlung bestimmter metabolischer Störungen, 0/1-kodierte Indikatorvariable, 1 falls folgende ATC-Kodes in Daten zu Arzneiverordnungen aus Apotheken: A16AB07, A16AA05, A16AX03, A16AX09, A16AB02, A16AB10, A16AX06, A16AX10, A16AB03, A16AB04, A16AX14, A16AB05, A16AB09, A16AB08, A16AB19, A16AX07, A16AX05, A16AX12, M01CC01, A16AX04, A16AB12, B02AB02, A16AB13, A05AA, C10AC, D02BB, A16AX, B06AB, P01BA
Ind_Haemophilie [0/1]	0,01% (0,01%)	Behandlung der Hämophilie, 0/1-kodierte Indikatorvariable, 1 falls folgende ATC-Kodes in Daten zu Arzneiverordnungen aus Apotheken: B02BD01, B02BD02, B02BD03, B02BD04, B02BD06, B02BD08, B02BD09, B02BD12, B02BD14, B02BD15, B02BD16, B02BD17, B02BD28, B02BD29, B02BD31, B02BD32, B02BD33, B02BD34, B02BD35, B02BD36, B02BD37, B02BD38, B02BD39, B02BD41, B02BD42, B02BD43, B02BX06
Ind_Theraneub [0/1]	0,21% (0,21%)	Neuartige Behandlung, 0/1-kodierte Indikatorvariable, 1 falls folgende ATC-Kodes in Daten zu Arzneiverordnungen aus Apotheken: L01FA, L01FB, L01FC, L01FD, L01FE, L01FF, L01FG, L01FX, L01EA, L01EB, L01EC, L01ED, L01EE, L01EF, L01EG, L01EH, L01EJ, L01EK, L01EL, L01EM, L01EN, L01EX, L04AX02, L04AX04, L04AX06

Angaben zu Anteilen von Betroffenen mit Angaben>0 bezogen auf n=7 070 986 Versicherte in Trainingsdaten (in Klammern: Angaben bezogen auf n=1 767 155 Versicherte in Testdaten), sämtliche Kodes wurden stets inklusive aller ggf. vorhandener Schlüssel auf weiter differenzierten Ebenen berücksichtigt, Ergebnisse ungewichtet.

### Gesamtbehandlungskosten, Annualisierung und Gewichtung

Zur Ermittlung von Gesamtbehandlungskosten wurden alle mit Versichertenbezug im W-DWH verfügbaren Informationen zu Kosten genutzt. Kosten der ambulanten (kassen-)ärztlichen und psychotherapeutischen Versorgung gem. § 295 SGB V wurden unter Zugrundelegung des Orientierungspunktwertes (2022: 11,2662 Cent; 7 ) aus fallbezogen ausgewiesenen Punktsummen sowie ggf. vorhandenen Angaben zu Euro-Beträgen geschätzt. Bei Behandlungen in Krankenhäusern gem. § 301 SGB V wurden fallbezogen ausgewiesene Kosten berücksichtigt, bei Arzneimitteln aus Apotheken gem. § 300 SGB V Bruttobeträge. Über diese drei Hauptleistungsbereiche (HLB) hinaus wurden ggf. auch Kosten von Heil- und Hilfsmitteln sowie Abrechnungen von Hebammen, Hospizen sowie zu ambulanten Behandlungen in Krankenhäusern sowie GKV-finanzierten Reha-Maßnahmen berücksichtigt. Negative Kostenangaben im Sinne von Erstattungen blieben unberücksichtigt.

Um unterjährig Versicherte adäquat berücksichtigen zu können, wurden – vergleichbar wie beim Morbi-RSA – Kosten stets in annualisierter Form betrachtet. Zugleich wurden Personen bei Analysen entsprechend ihrer anteiligen Versicherung innerhalb des Jahres 2022 gewichtet. So wird z. B. eine 2022 nur 183 Tage versicherte Person mit dem Gewicht 0,5 berücksichtigt, wobei Kosten zur Annualisierung mit dem Faktor 2 multipliziert sind, was sinngemäß der Extrapolation der dokumentierten Kosten auf ein ganzes Jahr entspricht. Gewichtete ermittelte Kostensummen zu Versichertengruppen entsprechen bei diesem Vorgehen dann den Summen der tatsächlich dokumentierten Kosten. Nach gewichteten Auswertungen lagen Gesamtkosten in Trainingsdaten bei durchschnittlich 3332 Euro (Standardabweichung SD 12 125), bei 5,44% der Versicherten waren keine Kosten dokumentiert (TeD: 3326 Euro (SD 12 367), 5,46% ohne dokumentierte Kosten).

### Gruppierungsschritte – Übersicht

Tab. 2 listet die Teilschritte zur exklusiven Gruppierung von Versicherten und resultierende Gruppierungen. Aufbauend auf Morbiditätsinformationen aus ZKGs sowie weiteren Merkmalen wird eine Population in 6 Teilschritten auf 4 hierarchischen Ebenen jeweils eindeutig exklusiven Gruppen zugeordnet. Einen Überblick liefert Abb. 1 . Jede Person ist in einem Jahr damit auf allen 4 Ebenen immer genau einer Gruppe zugeordnet, untergeordnete Gruppen sind jeweils nur einer Gruppe auf höher(en) Ebene(n) zugeordnet. Aufteilungen finden stets innerhalb von ggf. bereits zuvor gebildeten Gruppen, und dabei mit Rückgriff auf teils speziell eingegrenzte Merkmale, statt. Im Schritt 7 werden PGs (und damit auch jeweils zugeordnete Versicherte) ergänzend – und unabhängig von Zuordnungen auf übergeordneten Hierarchieebenen – 10 Kostenstrata zugeordnet. Die Schritte 1 bis 7 werden – nach grundlegenden methodischen Erläuterungen – in ihrer Abfolge noch näher im Abschnitt Ergebnisse beschrieben.

**Abb. 1:**
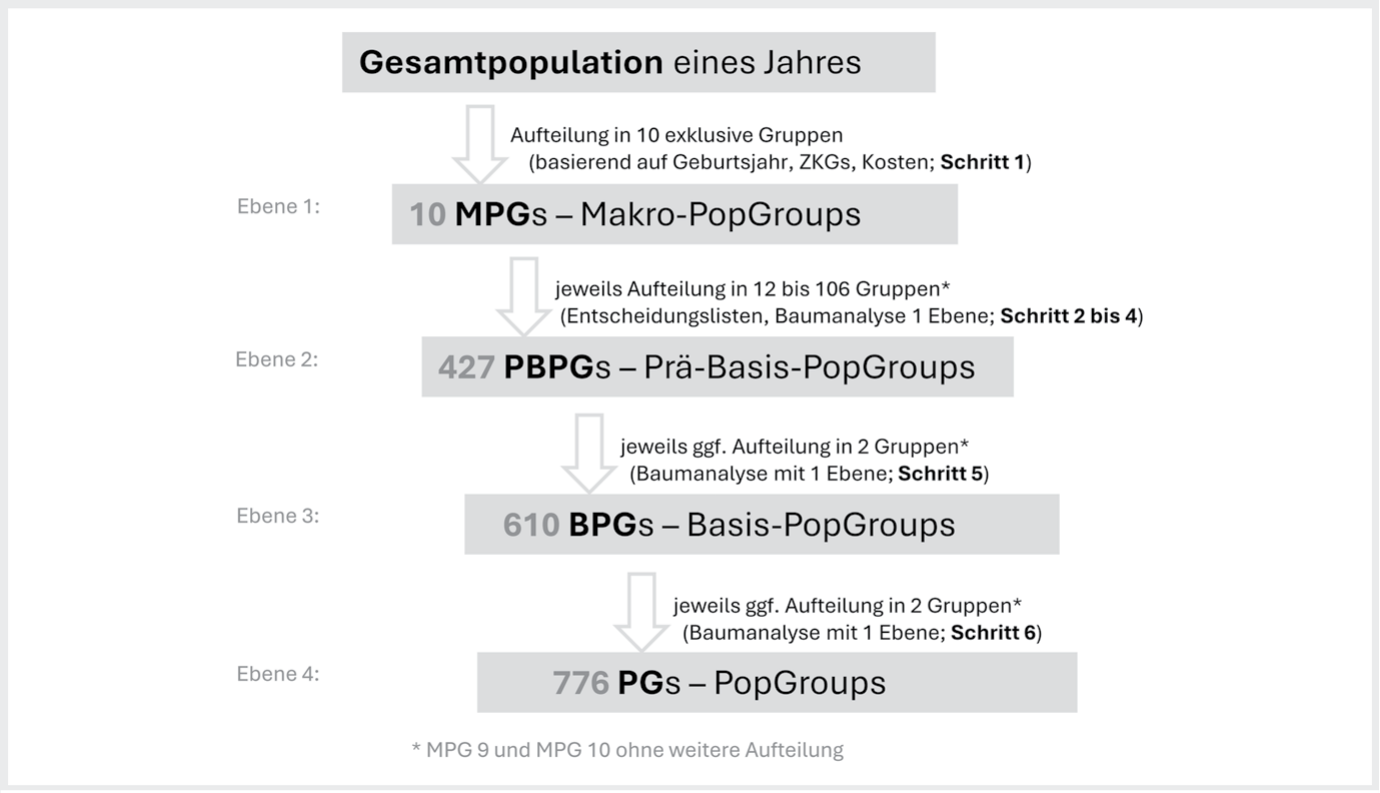
Gruppierung von Versicherten auf 4 hierarchischen Ebenen in 6 Schritten – PopGrouper 1.0. Erläuterungen zu den einzelnen Gruppierungsschritten 1 bis 6 sind der Ergebnisdarstellung zu entnehmen.

**Table:** **Tab. 2** Gruppierungen von Versicherten auf verschiedenen Ebenen – PopGrouper 1.0.

Schritt	Ebene	Kürzel	Anzahl	Bezeichnung (Verfahren), Grundlage; ggf. resultierende Regeln
1	1	**MPG**	10	**Makro-PopGroup** (Zuordnung regelbasiert, vorrangig inhaltlich begründet), basierend auf Geburtsjahr, ZKGs, Kosten
2	Zwischenschritte zur Ermittlung von 300 FE-ZKG-Kombinationen			**Führende Erkrankungen FE** (1. Entscheidungsliste), basierend auf ZKGs, Kosten; Regeln zur Identifikation von FEs
3				**Kombinationen** (1. Baumanalyse), basierend auf FEs, ZKGs, Kosten; Regeln zur Bildung von Kombinationen
4	2	**PBPG**	427	**Prä-Basis-PopGroup** (2. Entscheidungsliste), basierend auf ZKGs, Kombinationen, Kosten; Regeln zur PBPG-Bildung
5	3	**BPG**	610	**Basis-PopGroup** (2. Baumanalyse), basierend auf PBPGs, Zählvariablen zu MKGs, Kosten; Regeln zur BPG-Bildung
6	4	**PG**	776	**PopGroup** (3. Baumanalyse), basierend auf BPGs, weitere Merkmale gem. Tab. 1 , Kosten; Regeln zur PG-Bildung
7	K	**PGK**	10	**PopGroup Kostenstratum** (Zuordnungsregel), basierend auf Durchschnittskosten, Besetzungen von PGs; Regeln zur PGK-Bildung

Anmerkungen: Informationen zu ZKGs inklusive von direkt ZKG-assoziierten Informationen; Angaben zu Kosten ausschließlich zur Entwicklung des PopGroupers erforderlich, zur Nachbildung wird lediglich die Information benötigt, ob Kosten im Jahr angefallen sind oder nicht. Die Nummerierung der beschriebenen Schritte bezieht sich hier auf die exklusive Gruppierung. Sie weicht insofern von Nummerierungen bei Gesamtdarstellungen zum PopGrouper ab, in denen unter Anderem zwei Schritte zur Diagnosegruppierung vorangestellt sind.

### Entscheidungslisten, Baumanalysen sowie verwendete Kriterien

Für die Bildung von Teilpopulationen wurden im Rahmen der PopGrouper-Entwicklung jeweils mehrfach zwei algorithmische Vorgehensweisen genutzt (s. Tab. 2 ), die sich dem maschinellen Lernen (ML) zuordnen lassen und hier in Kurzform als Entscheidungslisten und Baumanalysen benannt sind. Aufgrund der spezifischen Anforderungen wurden beide Algorithmen als SAS-Programme speziell für den PopGrouper implementiert.

**Entscheidungslisten** können als spezielle Form von Entscheidungsbäumen gelten, bei denen ggf. jeweils nur einer von zwei zuvor entstandenen Zweigen anhand der Ausprägungen eines algorithmisch selektierten Merkmals weiter aufgeteilt wird 8 . Mit dem hierfür implementierten Algorithmus wird in jedem Durchlauf das Merkmal identifiziert (z. B. eine bestimmte ZKG-Zuordnung), nach dem dann betrachtete Versicherte in eine Gruppe mit den höchsten durchschnittlichen Kosten (z. B. Versicherte mit Hämophilie) sowie Übrige (ohne Hämophilie) aufgeteilt werden können, wobei beide Teilgruppen stets eine Mindestzahl an Versicherten und eine Grenze für gruppenbezogene Kosten nicht unterschreiten dürfen (s. weiter unten beschriebene Kriterien). Die weitere Aufteilung der nach Abspaltung der jeweils teuersten Teilgruppe verbleibenden Population wurde so lange wiederholt, bis Kriterien für weitere Teilungen nicht mehr erfüllt waren, wobei dann noch verbleibende Versicherte ggf. einer Restgruppe zugeordnet wurden. Der Algorithmus wurde so implementiert, dass Teilungsoptionen auch für eine hohe Zahl an potenziell teilungsrelevanten Merkmalen automatisiert geprüft werden können.

Die algorithmische Implementierung der (Entscheidungs-) **Baumanalysen** unterscheidet sich von der bei Entscheidungslisten dahingehend, als dass formal möglichst große Differenzen durchschnittlicher Kosten in zwei resultierenden Gruppen angestrebt werden und insbesondere dadurch, dass weitere Aufteilungsmöglichkeiten aller zuvor entstandenen Zweige geprüft werden, bis Kriterien für weitere Aufteilungen nicht mehr erfüllt sind oder eine vorgegebene max. Zahl an Teilungsebenen erreicht ist (die im Rahmen der Analysen zum PopGrouper 1.0 auf eine Ebene begrenzt wurde). Zudem wurde der Algorithmus für Baumanalysen so implementiert, dass neben dichotomen (0/1-kodierten) Merkmalen auch ordinal sowie metrisch skalierte Merkmale hinsichtlich ihrer Teilungsrelevanz und -möglichkeiten geprüft und berücksichtigt werden können. Für die Analyse zum PopGrouper 1.0 wurden jedoch ausschließlich gruppierte metrische Merkmale (Alter, Beatmungsstunden) berücksichtigt, um die Zahl der Grenzwerte im resultierenden Regelwerk zu limitieren.

Bei Anwendung beider Algorithmen wurde vor Teilungen im Sinne einheitlich festgelegter **Kriterien** stets sichergestellt, dass in Trainingsdaten Subgruppen mit min. 25 Personen und Gesamtkosten in Höhe von min. 8 784 026 Euro resultierten, womit entstandene Gruppen nach Hochrechnung auf die Bevölkerung Deutschlands approximativ stets für jährliche Kosten in Höhe mehr als 100 Mio. Euro verantwortlich sein sollten. Bei Aufteilungen in Baumanalysen wurde zudem gefordert, dass sich durchschnittliche Gesamtkosten in zwei Subgruppen um min. 250 Euro sowie relativ um min. 25% unterschieden.

### Kostendiskrimination – MSE, MAE

Die Validierung der entwickelten Gruppierungen erfolgte primär auf Basis der Testdaten zum Jahr 2022. Gruppenzuordnungen wurden mit aus Trainingsdaten gewonnen Regeln in Testdaten nachgebildet, um mittlere quadratische Abweichungen (mean squared error, MSE) sowie mittlere absolute Abweichungen (mean absolute error, MAE) bei zunehmender Gruppierungstiefe als maßgebliche Kennzahlen zur Beurteilung der Klassifikationsgüte darzustellen. Ergänzend werden die Kennzahlen auch für die zur Entwicklung genutzten Trainingsdaten 2022 sowie für die nach PopGrouper-Regeln gruppierten Gesamtdaten zu den Jahren 2021 und 2023 dargestellt.

## Ergebnisse

### Makro-PopGroups (MPGs) – Schritt 1 – Ebene 1

Auf einer übergeordneten Ebene 1 wurde jeder Versicherte genau einer von 10 MPGs zugeordnet. Die Definition der MPGs orientiert sich an international bereits existierenden Groupern. Neugeborene (MPG01) und Schwangere (MPG02) bilden 2 erste, exklusive Gruppen, da bei diesen Versicherten mit sehr spezifischen Bedarfen zu rechnen ist. Weitere MPG-Zuordnungen basieren vorrangig auf versichertenbezogen zugeordneten ZKGs und deren Schweregraden, womit 8 weitere Gruppen von – vereinfacht formuliert – „sehr kostenintensiv/krank“ bis „ohne Kosten/ohne Erkrankungshinweise“ resultieren. Personen ohne jegliche Erkrankungshinweise in Form einer ZKG wird eine Pseudo-ZKG zugeordnet, welche technisch als Merkmal bei weiteren algorithmischen Aufteilungen von Gruppen benötigt wird. Zur MPG-Zuordnung erforderlich sind die Merkmale Geburtsjahr, potenziell 433 ZKG-Zuordnungen und bei Versicherten ohne reguläre ZKG-Zuordnung (bzw. mit Pseudo-ZKG) die Information, ob Kosten dokumentiert sind oder nicht. Die Zuordnung zu MPGs erfolgt hierarchisch entsprechend der Abfolge der MPG-Nummern (s. Tab. 3 ) – ist ein Versicherter in einem Schritt einer MPG zugeordnet, gilt stets genau diese Zuordnung.

**Table:** **Tab. 3** Zuordnung von Versicherten zu 10 Makro-PopGroups (MPGs).

MPG	Bezeichnung (Populations- sowie Kostenanteile)*	Selektionsregel, Erläuterungen
01	Neugeborene (1,1%, 1,2%)	alle im Beobachtungsjahr oder im Vorjahr geborene Versicherte (unabhängig von der Morbidität)
02	Schwangerschaft, Geburt, Wochenbett (1,1%, 1,7%)	alle verbliebenen Versicherten mit Dokumentation mindestens einer von 8 ZKG aus der MKG Z20 „Schwangerschaft, Geburt und Wochenbett“ (Z2001 bis Z2008)
03	Schwerwiegende Hochkostenfälle (2,8%, 25,3%)	alle verbliebenen Versicherten mit Dokumentation mindestens einer von 57 ZKG mit Schweregrad-Einstufung „sehr schwer“ und zugleich sehr hohen ZKG-assoziierten Ausgaben
04	Aktiv behandelte bösartige Neubildungen (1,1%, 6,1%)	alle verbliebenen Versicherten mit Dokumentation mindestens einer von 27 ZKG mit Zuordnung zu aktiv behandelten Krebserkrankungen
05	Mindestens eine (sehr) schwere Erkrankung (10,5%, 23,6%)	alle verbliebenen Versicherten mit Dokumentation von mindestens einer von insgesamt 173 ZKG mit Schweregrad-Einstufung „schwer“ oder „sehr schwer“
06	Mindestens eine moderate Erkrankung (22,8%, 23,5%)	alle verbliebenen Versicherten mit Dokumentation von mindestens einer von insgesamt 86 ZKG mit Schweregrad-Einstufung „moderat“
07	Mindestens eine leichte Erkrankung (21,9%, 13,8%)	alle verbliebenen Versicherten mit Dokumentation von mindestens einer von insgesamt 87 ZKG mit Schweregrad-Einstufung „leicht“
08	Mindestens eine sehr leichte Erkrankung (17,2%, 3,9%)	alle verbliebenen Versicherten mit Dokumentation von mindestens einer von insgesamt 87 ZKG mit Schweregrad-Einstufung „sehr leicht“
09	Inanspruchnahme ohne Erkrankung (16,1%, 1,0%)	Versicherte ohne ZKG-Zuordnung, jedoch mit versichertenbezogen dokumentierten Kosten (ausschließlich mit Pseudo-ZKG Z0000)
10	Keine Inanspruchnahme (5,4%, 0,0%)	Versicherte ohne ZKG-Zuordnung ohne versichertenbezogen dokumentierte Kosten (ausschließlich mit Pseudo-ZKG Z0000)

^*^ gewichtete Anteile in Trainingsdaten 2022 zu (roh) n=7 070 986 Versicherten

### Ermittlung führender Erkrankungen (FEs) – Schritt 2

Bei Analysen innerhalb der MPGs 01 bis 08 wurden Entscheidungslisten genutzt, um führende Erkrankungen (FEs) im Sinne von ZKGs oder führende Erkrankungsgruppen (MKGs und schwere MKGs bei Zuordnungen von (sehr) schweren ZKGs) mit besonders hohen Kosten zu identifizieren. Im Ergebnis wurden so innerhalb der MPGs alle Personen genau einer vorrangigen Erkrankungs- sowie ggf. zuletzt einer Restgruppe zugeordnet, wobei Personen in nachfolgend gebildeten FEs stets geringere durchschnittliche Kosten als vorausgehend abgegrenzte FEs aufweisen. Unter Einhaltung der im Abschnitt Methoden beschriebenen Kriterien konnten Personen in diesem Schritt ggf. jeweils einer von insgesamt 300 unterschiedlichen FEs zugeordnet werden. Innerhalb von MPGs wurde dabei auf unterschiedlich eingegrenzte Informationen zurückgegriffen (s. Tabelle A1 im Anhang), wobei zwischen 12 und 65 Gruppen mit bestimmten FEs gebildet wurden.

### Identifikation von (Erkrankungs-)Kombinationen – Schritt 3

Basierend auf in Schritt 2 identifizierten FEs sowie erneut erkrankungsbezogenen Informationen in Form von ZKG- sowie MKG-Zuordnungen (s. Tabelle A2) wurden relevante Erkrankungskombinationen identifiziert. Für jede FE wurde dazu in einer Baumanalyse geprüft, welche Kombination unter Einhaltung von Kriterien für Baumanalysen ggf. zu einer Aufteilung in zwei Teilgruppen mit der höchsten Kostendifferenz führte. Insgesamt konnten so 163 Kombinationen identifiziert werden. Innerhalb der einzelnen MPGs wurden zwischen zwei (MPG08) und 47 (MPG05) Kombinationen gebildet (s. **Tabelle A3** ). Kombinationen mit „Restgruppen“ blieben stets unberücksichtigt.

### Prä-Basis-PopGroups (PBPGs) – Schritt 4 – Ebene 2

Zur Identifikation von PBPGs mittels einer Entscheidungsliste wurde auf 163 Indikatorvariablen zu zuvor identifizierten Kombinationen sowie erneut auf Informationen in Form von ZKGs und daraus ableitbaren Erkrankungsgruppen zurückgegriffen. Das Vorgehen entspricht damit dem Schritt 2 mit Erweiterung der Liste potenziell teilungsrelevanter Variablen um die zuvor identifizierten Kombinationen. In den einzelnen MPGs resultierten dabei zwischen 12 und 106 PBPGs, insgesamt wurden 427 PBPGs abgegrenzt (s. Tabelle A4).

### Basis-PopGroups (BPGs) – Schritt 5 – Ebene 3

Die Bildung der BPGs erfolgte anschließend ggf. mit Aufteilung von PBPGs in einer Baumanalyse mit max. einer Teilung ausschließlich anhand einer von zwei Zähvariablen, nämlich der Anzahl der unterscheidbaren MKGs im Allgemeinen oder der Anzahl der unterscheidbaren MKGs mit Zuordnungen (sehr) schwerer ZKGs. Der PopGrouper 1.0 umfasst nach diesem Schritt insgesamt n=610 disjunkte Populationssubgruppen im Sinne von BPG.

### PopGroups (PGs) – Schritt 6 – Ebene 4

Während bis zur Ebene der BPG-Bildung weitgehend ausschließlich auf Informationen zurückgegriffen wird, die aus ZKGs herleitbar sind, wurden bei der nachfolgenden Aufteilung der BPGs in einer Baumanalyse in jeweils max. zwei PGs neben Geschlecht und Altersgruppen (in Anlehnung an die im Morbi-RSA verwendeten Altersgrenzen) die sieben in Tab. 1 gelisteten Merkmale berücksichtigt. Es resultierte eine finale Aufteilung der Population auf 776 PGs.

### PopGroup Kostenstrata (PGKs) – Schritt 7 – Ebene K

Die PGs wurden abschließend anhand durchschnittlicher Gesamtkosten von zugeordneten Versicherten (unabhängig von übergeordneten Gruppierungen) jeweils einem von insgesamt 10 Kostenstrata zugeordnet. PGK01 umfasst PGs mit den höchsten und PGK10 diejenigen PGs mit den geringsten Kosten. Den PGKs bzw. zugeordneten Versicherten sollte dabei möglichst etwa 1/10 der beobachteten Gesamtausgaben zuschreibbar sein. Aufgrund der begrenzten Zahl an Teilungsmöglichkeiten nach PGs variieren die gewichteten Anteile in Trainingsdaten de facto von 9,5% bis 10,7%. Populationsanteile sind für Trainings- und Testdaten in Abb. 2 links und rechts der zentralen Achse durch Balken dargestellt, real zugeordnete Kostenanteile werden in Klammern angegeben.

**Abb. 2:**
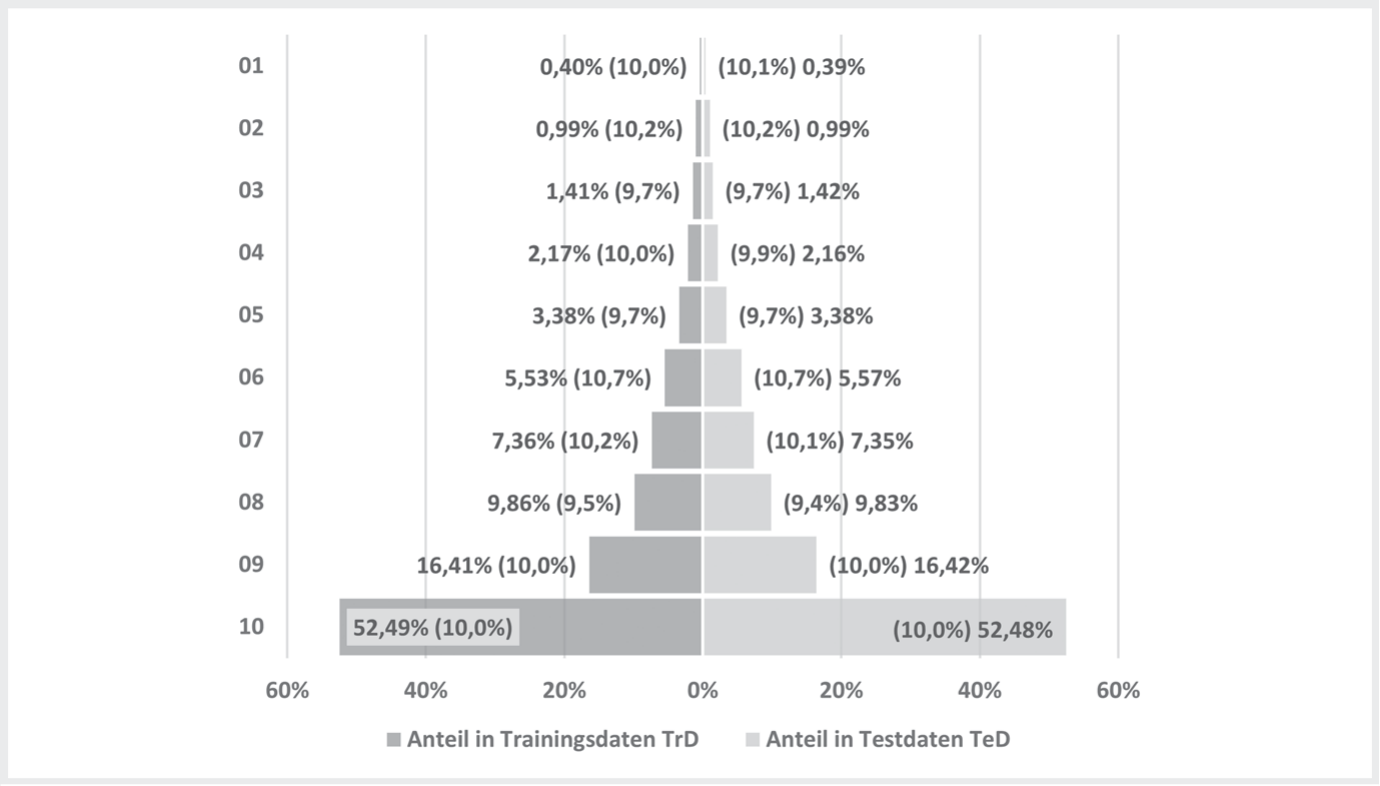
PopGroup Kostenstrata (PGKs) 01 bis 10 – effektiv zugeordnete Populations- und Kostenanteile in Trainings- und Testdaten 2022. Populationsanteile sowie Gesamtkostenanteile (in Klammern), gewichtet, Ergebnisse nach Trainings- und Testdaten zu (roh) n=7 070 986 bzw. n=1 767 155 Versicherten.

### Kennzahlen zur Kostendiskrimination von Gruppierungen

MSE-Werte, die hier aufgrund der Populationsgröße nahezu vollständig Stichprobenvarianzen entsprechen, reduzierten sich in Testdaten zum Jahr 2022 mit einer PG-Gruppierung auf 61,2% des Wertes in der nicht gruppierten Population (s. Tab. 4 ), womit PGs in Testdaten den (komplementären) Anteil von 38,8% der beobachteten Kostenvarianz erklärten. Der erklärte Varianzanteil lag auf unterschiedlichen Gruppierungsebenen ca. 0,3 Prozentpunkte niedriger, sofern Versicherte ohne dokumentierte Kosten aus der MPG10 bei Analysen unberücksichtigt blieben (s. Tabelle A5). MAE-Werte wurden anteilig mit zunehmender Gruppierungstiefe stets stärker als MSE-Werte reduziert.

**Table:** **Tab. 4** Kennzahlen zur Diskrimination von Gesamtkosten durch unterschiedliche Gruppierungsebenen – PopGrouper 1.0.

	mean squared error – MSE [%]						mean absolute error – MAE [%]				
	TrD	TeD		Gesamtpop.			TrD	TeD	Gesamtpop.		
Erhebungsjahr	2022		2022		2021	2023	2022	2022		2021	2023
*Anzahl Versicherte (in Tsd., roh)*	*7071*		*1767*		*9165*	*8772*	*7071*	*1767*		*9165*	*8772*
*MSE in Mio. €* ^*2*^ *; MAE in €* ^*2*^	*152,31*		*158,43*		*152,13*	*172,18*	*4041*	*4035*		*3858*	*4366*
Population insgesamt	100,0		**100,0**		100,0	100,0	100,0	**100,0**		100,0	100,0
Makro-PopGroups (MPGs)	81,6		**82,4**		82,4	80,6	71,0	**71,2**		69,7	69,2
Führende Erkrankungen (FEs)	69,8		**70,2**		71,0	69,7	61,6	**61,7**		61,4	61,4
Prä-Basis-PopGroups (PBPGs)	67,3		**67,9**		68,8	67,2	60,1	**60,2**		60,0	59,9
Basis-PopGroups (BPGs)	65,9		**66,4**		67,4	65,8	59,3	**59,4**		59,2	59,1
PopGroups (PGs)	61,4		**61,2**		62,7	61,2	57,7	**57,7**		57,4	57,5
PopGroup Kostenstrata (PGKs)	67,4		**68,0**		68,7	67,4	61,0	**61,1**		61,0	60,9

gewichtete Berechnungen zu annualisierten Gesamtkosten, nach Gruppierung verbleibende MSE- bzw. MAE-Anteile jeweils bezogen auf MSE- bzw. MAE-Werte in der nicht gruppierten Population

## Diskussion

Mit der Entwicklung des PopGroupers und der finalen, jahresbezogenen Zuordnung jedes Versicherten zu genau einer PG sollten möglichst viele der einleitend gelisteten Ziele erreicht werden. Eine erste Aufteilung der Population auf 10 MPGs erfolgte nach Sichtung umfangreicher Voranalysen vorrangig auf Basis medizinisch-inhaltlicher Überlegungen, was insbesondere für die gesonderte Betrachtung von Neugeborenen (MPG01) sowie von Schwangeren (MPG02) mit absehbar spezifischen Bedarfen gilt. Die Ableitung weiterer MPGs basiert auf den in Arbeitsschritt I operationalisierten Zuordnungen von ZKGs mit Schweregraden und MKGs 6 , woraus eine erste Gliederungsebene resultiert (s. Tab. 3 ).

### Algorithmische Aufteilung

Weiter Aufteilungen der MPGs erfolgten algorithmisch (s. Tab. 2 ). Im Hinblick auf die Anwendbarkeit des Groupers – beispielsweise für Versorgungsstrukturplanungen – erscheint dabei die Bildung möglichst homogener Gruppen gem. Ziel Z1 von prioritärer Bedeutung. Bestimmte klassische statistische Verfahren wie Clusteranalysen ermöglichen zwar die Identifikation homogener Subgruppen in Populationen 9 , auch in anderen Populationen anwendbare Gruppierungsregeln lassen sich aus den Ergebnissen jedoch nicht direkt ableiten. In der Projektkonzeption war zur Gruppierung von Versicherten, wie schon bei der Entwicklung anderer Grouper 1 2 3 , der Einsatz des Classification and Regression Tree (CART) Algorithmus vorgesehen 10 . Dieser spaltet Populationen schrittweise binär in Zweige von Entscheidungsbäumen auf, wobei zugleich ein Regelwerk resultiert, das sich für Abgrenzungen auch in anderen Populationen nutzen lässt. Entscheidungsbäume werden häufig als gut nachvollziehbar beschrieben, was bei Gegenüberstellungen mit anderen ML-Techniken bzw. Algorithmen der künstlichen Intelligenz (KI) gerechtfertigt erscheint. Bei einer größeren Zahl an Teilungsebenen resultieren bei Entscheidungsbäumen jedoch lange Verkettungen von Bedingungen, die eine kurze und nachvollziehbare Beschreibung der gebildeten Gruppen auch hier unmöglich machen (z. B. Vorhandensein von Diagnosen aus Gruppe A und nicht B und nicht C und D und E und nicht F usw.).

Die beschriebene Problematik bildete den maßgeblichen Grund, zur algorithmischen Aufteilung von Populationen Entscheidungslisten sowie Entscheidungsbäume mit Begrenzung auf eine Teilungsebene zu nutzen. Mit Entscheidungslisten werden Versicherte primär einer Erkrankung(sgruppe) zugeordnet, wodurch die Beschreibung von Gruppen erheblich erleichtert wird. Bedacht werden muss allerdings, dass nicht alle Personen mit entsprechenden Erkrankungen dieser einen Gruppe zugeordnet werden, sondern diese Erkrankungen als „nachrangig bedeutsam“ auch in zuvor selektierten (teureren) Gruppen vorkommen können. Mit der jetzigen Implementierung und Anwendung von Entscheidungslisten wird zudem implizit unterstellt, dass diejenige Gruppenzuordnungen, für die bei zulässigen Teilungen die höchsten durchschnittlichen Gesamtkosten ermittelt werden, auch führende bzw. maßgebliche Erkrankung(sgrupp)en der zugeordneten Versicherten charakterisieren. Wären beispielsweise einer Person die ZKGs „Atherosklerose“, „Arrhythmie“, „Störung des Fettstoffwechsels“ und „Zerebrale Blutung oder Hirninfarkt“ zugeordnet, dann wäre bei 4-maliger Aufteilungen einer Population anhand jeweils eines der Merkmale zu erwarten, dass für die Subgruppe mit „Zerebrale Blutung oder Hirninfarkt“ die höchsten durchschnittlichen Kosten ermittelt werden, womit diese dann als führende Erkrankung in einer Entscheidungsliste zugeordnet würde und die verbleibenden 3 Merkmale i.d.R. nicht explizit zur Charakterisierung herangezogen würden. Nach stichprobenartiger Sichtung resultierten aus dem algorithmischen Vorgehen bei Entscheidungslisten augenscheinlich zumeist gut nachvollziehbare Ergebnisse, eine systematische Validierung war jedoch nicht leistbar. Eher unscharfe Zuordnungen sind – auch aus klinischer Sicht – insbesondere bei Personen mit ausschließlicher Zuordnung von (sehr) leichten Erkrankungen zu erwarten.

### Gliederung, Abfolgen und Elemente von Zuordnungsschritten

Als sehr hilfreich für die algorithmischen Teilungen von Gruppen erwiesen sich die Gruppierungen der ZKGs in übergeordneten MKGs sowie auch die ZKG-bezogen ermittelten Schweregrade. Mit den MKGs konnten übergeordnete Erkrankungsgruppen berücksichtigt werden, wenn von einzelnen ZKGs für die Bildung einer FE, Kombination oder PBPG zu wenige Versicherte betroffen waren.

Um gem. Ziel Z5 explizit auch relevante 2er-Kombinationen benennen zu können, wurden die Schritte 3 und 4 in der Entwicklung des PopGroupers implementiert. Andernfalls hätte Schritt 5 direkt auf Schritt 2 folgen können (s. Tab. 2 ). Die Berücksichtigung von Kombinationen erscheint dabei auch durch die damit verbundene Abnahme der nicht erklärten Varianz legitimiert (s. Tab. 4 ). Darüber hinaus wurden zur Bildung von BPGs aus PBPGs noch ZKG-basierte Zählvariablen berücksichtigt.

Eine Berücksichtigung von außerhalb von GKV-Daten nur begrenzt verfügbaren Merkmalen erfolgte erst in Schritt 6 bei der Differenzierung von PGs, womit die übergeordneten BPGs in Übereinstimmung mit Ziel Z6 noch ohne die Merkmale gem. Tab. 1 gebildet werden können.

### Eigenschaften der gebildeten Gruppierungen

Die Entwicklung des Groupers zielte primär auf die Bildung möglichst homogener Gruppen hinsichtlich des Erkrankungsspektrum sowie gesundheitlicher Versorgungsbedarfe ab. Die Homogenität von Gesundheitskosten ist dabei nur eines der möglichen und relevanten Kriterien, erschien jedoch bei einer Berücksichtigung des gesamten Erkrankungsspektrums im Rahmen der Grouper-Entwicklung weitgehend alternativlos. Trotz eines Fokus auch auf klinisch-inhaltlich nachvollziehbare Gruppenbildungen bei der Entwicklung, ließ sich in Testdaten zum Jahr 2022 38,8% der Kostenvarianz durch PG-Zuweisungen erklären (womit der erklärte Anteil in Testdaten etwas höher als in Trainingsdaten liegt). Offensichtlich lassen sich dabei in Trainings- und Testdaten, anders als in manchen Beispielen zu ML-Techniken, sehr ähnliche Ergebnisse ermitteln, was aus der hohen Zahl an Beobachtungen bei hier dargestellten Analysen resultieren dürfte (s. Tab. 4 sowie Abb. 2 ). Positiv lässt sich zudem hervorheben, dass sich nach Anwendung von Gruppierungsregeln des PopGroupers 1.0 in den Jahren 2021 und 2023 vergleichbare Eigenschaften wie 2022 zeigten, wobei auf nahezu allen Gruppierungsebenen die besten Ergebnisse überraschend mit Daten zum Jahr 2023 resultierten. Dass im Jahr 2023 bessere Kennwerte als für das Entwicklungsjahr resultieren, könnte damit zusammenhängen, dass die Versorgung 2022 noch stärker von der Coronapandemie beeinflusst war, wobei COVID-19-Infektionen und direkt assoziierte Folgen im Grouper nach Herleitung aus drei Diagnosegruppen des Morbi-RSA (DxG) nur ansatzweise abgebildet werden. Daten zu 2023 standen für eine Entwicklung des Groupers in der Projektlaufzeit erst zu spät zur Verfügung. Ergänzend sei an dieser Stelle darauf hingewiesen, dass allen gebildeten Gruppierungen im Zuge der Entwicklung auch gruppenbezogene Schweregrade sowie Relativgewichte zugewiesen wurden, welche die Nutzungsmöglichkeiten des Groupers erweitern. Da diese Kennwerte für die Herleitung der Gruppierung unerheblich sind, wird hier nicht näher auf Ermittlung, Eigenschaften und Nutzungsmöglichkeiten eingegangen. Zugleich muss auch eine umfassendere Bewertung von Grouper-Eigenschaften nachfolgenden Publikationen vorbehalten bleiben.

### Limitationen

- Daten zum Jahr 2022 könnten aufgrund von Besonderheiten im Verlauf der Coronapandemie nicht ideal für die Grouper-Entwicklung gewesen sein.
- Einschränkungen bedingt durch eine Beschränkung der Analysen auf Daten einer Krankenkasse lassen sich nicht ausschließen.
- Umgekehrt muss betont werden, dass – selbst im Falle einer rechtzeitigen Verfügbarkeit von Zugriffsmöglichkeiten auf GKV-übergreifende Daten im Forschungsdatenzentrum Gesundheit (FDZ) – die Entwicklung des PopGroupers bereits vor dem Hintergrund der bislang angedachten Beschränkungen von Ergebnismengen mit FDZ-Zugriffen nicht realisierbar gewesen wäre. Im Projektverlauf wurden Ergebnismengen gemeinschaftlich vom Team gesichtet, welche das angedachte FDZ-Limit um Zehnerpotenzen überschritten hätten.
- Aufgrund der inhaltlich-klinischen Fokussierung wurde eine „statistisch optimale“ Gruppierung weder angestrebt noch erreicht. Ein Teil der eingeschlagenen Wege lässt sich insofern nicht empirisch begründen.
- Der Rückgriff auf Erkrankungsinformationen in Form von DxG, die strikt gemäß Vorgaben zum Morbi-RSA nachgebildet wurden, erschwert die Nachbildung der hier vorgestellten Gruppierungen, sofern genau diese DxG-Zuordnungen in den jeweils verwendeten Daten nicht bereits verfügbar sind.

## Relevanz und Ausblick

Mit dem PopGrouper 1.0 liegt erstmals ein für GKV-Daten in Deutschland angepasster bevölkerungsbezogener Grouper vor, der umfassend dokumentiert ist und frei genutzt werden kann. Die zur Nachbildung des Groupers erforderlichen Schritte sind in einem Working paper detailliert beschrieben 11 . Ergebnisse zum PopGrouper 1.0 deuten auf gut nutzbare Gruppierungen für die Datenjahre 2021 bis 2023 hin, zu denen bereits vollständige Gruppierungsregeln existieren und bereitgestellt werden können. Anpassungen für Folgejahre sollten angestrebt werden. Dabei sollte möglichst auch geprüft werden, inwiefern Vereinfachungen des Groupers und seiner Pflege ohne Qualitätsverluste möglich sind. Erprobt werden sollte dabei auch, inwiefern eine ZKG-Bildung zur Gruppierung von Diagnosen ohne eine zunächst vollständige Nachbildung von DxG gemäß Regeln zum Morbi-RSA möglich ist, was die Pflege des Grouper erleichtern und den Grouper auch für eine Nutzung außerhalb Deutschlands attraktiver machen könnte.

## Fördermittel

Das dieser Veröffentlichung zugrundliegende Projekt wurde mit Mitteln des Innovationsausschusses beim Gemeinsamen Bundesausschuss unter dem Förderkennzeichen 01VSF20027 gefördert.

## Zusätzliches Material

Zusätzliches Material Zusätzliches Material
