# Supplementary material for: Formation of disease- and needs-related homogeneous groups of insured persons – PopGrouper 1.0
Source: Gesundheitswesen. 2026 Jun 19;88(10):619–28. [Article in German] doi: 10.1055/a-2872-0161 (PMC13645419; doi:10.1055/a-2872-0161)
Supplement: Supplementary file 1 [file 10-1055-a-2872-0161-2025-06-2290-oa.pdf]

Anhang (zur Online-Publikation)

Verwendete Abkürzungen

Abkürzung	Erläuterung
**BPGs**	Basis-PopGroups (PopGrouper 1.0: Gruppierung auf 3. Gliederungsebene mit insgesamt 610 disjunkten Populationsgruppen)
**CART**	Classification and Regression Tree - Algorithmus aus dem Bereich des maschinellen Lernens zur Aufteilung von Beobachtungen in exklusive Gruppen
**DxG**	Diagnosegruppe im Rahmen des morbiditätsorientierten Risikostrukturausgleichs (Morbi-RSA)
**FDZ**	Forschungsdatenzentrum Gesundheit (beim Bundesinstitut für Arzneimittel und Medizinprodukte)
**FEs**	Führende Erkrankungen (PopGrouper 1.0: führende Erkrankung im Sinne einer ZKG, effektiv eine von hier nur 300 unterschiedlichen ZKGs)
**GKV**	Gesetzliche Krankenversicherung
**HLB**	Hauptleistungsbereich - Ausgabenbereich im Rahmen des morbiditätsorientierten Risikostrukturausgleichs (Morbi-RSA)
**KI**	Künstliche Intelligenz
**MAE**	mean absolute error - Maßzahl für die Abweichungen der beobachteten von vorhergesagten Werten (weniger beeinflusst durch Ausreißer als MSE-Werte)
**MKG**	Makrokrankheitsgruppen (PopGrouper 1.0: Aufteilung von Krankheiten in 32 Erkrankungsgruppen)
**ML**	Maschinelles Lernen
**Morbi-RSA**	Morbiditätsorientierter Risikostrukturausgleich (zum Finanzausgleich zwischen den gesetzlichen Krankenversicherungen/Krankenkassen in Deutschland)
**MPGs**	Makro-PopGroups (PopGrouper 1.0: Gruppierung von Personen auf 1. Gliederungsebene mit insgesamt 10 disjunkten Populationsgruppen)
**MSE**	mean squared error - Maßzahl für die Abweichungen der beobachteten von vorhergesagten Werten, häufiger im Bereich des maschinellen Lernens verwendet
**PBPGs**	Prä-Basis-PopGroups (PopGrouper 1.0: Gruppierung von Personen auf 2. Gliederungsebene mit insgesamt disjunkten 427 Populationsgruppen)
**PGKs**	PopGroup Kostenstrata (PopGrouper 1.0: Aufteilung der Population in 10 Gruppen mit jeweils annähernd gleichen Kostenanteilen anhand von PGs und der durchschnittlichen Gesundheitskosten von PG-zugeordneten Personen)
**PGs**	PopGroups - voll ausdifferenzierte Aufteilung einer Population (PopGrouper 1.0: Gruppierung auf 4. Gliederungsebene mit 776 disjunkten Populationsgruppen)
**Pseudo-ZKG**	Merkmale, die sich aus der Zuordnung oder Nicht-Zuordnung von bestimmten MKGs oder ZKGs ableiten lassen, insbesondere auch die Pseudo-ZKG Z0000 mit Ausprägung = 1 bei Personen, denen ansonsten keinerlei Erkrankung im Sinne einer ZKG zugeordnet werden konnte
**SAS**	Software zur Aufbereitung und Analyse von Daten (Statistical Analysis System) des SAS-Instituts
**SD**	Standardabweichung
**SGB**	Sozialgesetzbuch
**TeD**	Testdaten - für maschinelles Lernen, hier Zufallsauswahl von 20% der Population
**TrD**	Trainingsdaten - für maschinelles Lernen, hier Zufallsauswahl von 80% der Population
**W-DWH**	Wissenschafts-Data Warehouse der BARMER
**ZKGs**	Zusammengefasste Krankheitsgruppen – Gliederung/Zuordnung aller berücksichtigter Diagnosen zu übergeordneten Diagnosegruppen (PopGrouper 1.0: 433 Gruppen)

Tabelle A1: Innerhalb von MPG genutzte ZKG sowie genutzte abgeleitete Merkmale

**MPG FE**	**Bezeichnung**	**genutzte ZKG und ggf. aus ZKG abgeleitete Merkmale**
**01** P0101-P0113	Neugeborene	9 ZKG nur aus der MKG 27, differenzierbare MKG 01 bis 32 basierend auf allen ZKG (dichotome Variablen/Merkmale ZMK01 bis ZMK32 mit Wert/Ausprägung = 1, falls MKG zugeordnet), zwei Pseudo-ZKG, die die Dokumentation (mindestens) einer von 57 ZKG aus der MPG 03 anzeigt (ZZMK3) oder die die Dokumentation (mindestens) einer von 27 ZKG aus der MPG 04 anzeigt (ZZMK4), Pseudo-ZKG ZREST für Restgruppe (der alle Personen der MPG zugeordnet werden)
**02** P0201-P0212	Schwangerschaft, Geburt, Wochenbett	8 ZKG nur aus der MKG 20, differenzierbare MKG 01 bis 32 basierend auf allen ZKG (ZMK01 bis ZMK32), zwei Pseudo-ZKG, die die Dokumentation (mindestens) einer von 57 ZKG aus der MPG 03 anzeigt (ZZMK3) oder die die Dokumentation (mindestens) einer von 27 ZKG aus der MPG 04 anzeigt (ZZMK4), Pseudo-ZKG ZREST für Restgruppe (der alle Personen der MPG zugeordnet werden)
**03** P0301-P0343	Schwerwiegende Hochkostenfälle	57 ZKG der MPG 03, 17 differenzierbare MKG basierend auf genau diesen ZKG (ZMK01 … ZMK25), Pseudo-ZKG ZREST für Restgruppe (der alle Personen der MPG zugeordnet werden)
**04** P0401-P0431	Aktiv behandelte bösartige Neubildungen	27 ZKG von Z0230 bis Z0256 der MPG 04, eine differenzierbare MKG 02 basierend auf genau diesen ZKG (ZMK02, der alle Personen der MPG zugeordnet werden), 26 Pseudo-ZKG (M0230 … M0256), sofern neben der ZKG Z0255 „Metastasen, aktiv behandelt" im selben Jahr noch eine entsprechende weitere ZKG der MPG 04 dokumentiert ist
**05** P0501-P0565	Mindestens eine (sehr) schwere Erkrankung	170 ZKG mit Schweregrad-Einstufung „schwer" oder „sehr schwer", differenzierbare MKG basierend auf genau diesen ZKG (ZMK01 … ZMK31), Pseudo-ZKG ZREST für Restgruppe (der alle Personen der MPG zugeordnet werden)
**06** P0601-P0660	Mindestens eine moderate Erkrankung	87 ZKG mit Schweregrad-Einstufung „moderat", differenzierbare MKG basierend auf genau diesen ZKG (ZMK01 … ZMK29), Pseudo-ZKG ZREST für Restgruppe (der alle Personen der MPG zugeordnet werden)
**07** P0701-P0743	Mindestens eine leichte Erkrankung	86 ZKG mit Schweregrad-Einstufung „leicht", differenzierbare MKG basierend auf genau diesen ZKG (ZMK01 … ZMK31), Pseudo-ZKG ZREST für Restgruppe (der alle Personen der MPG zugeordnet werden)
**08** P0801-P083127	Mindestens eine sehr leichte Erkrankung	87 ZKG mit Schweregrad-Einstufung „sehr leicht", differenzierbare MKG basierend auf genau diesen ZKG (ZMK01 … ZMK32), Pseudo-ZKG ZREST für Restgruppe (der alle Personen der MPG zugeordnet werden)
**09** P0901	Inanspruchnahme ohne Erkrankung	(ausschließlich mit Pseudo-ZKG Z0000)
**10** P1001	Keine Inanspruchnahme	(ausschließlich mit Pseudo-ZKG Z0000)

Tabelle A2: Für die Zuordnung zu Erkrankungskombinationen benötigte Variablen

Variablenname	Merkmal	Merkmalsausprägungen, -range und -beschreibung (ggf. Basiswert)
Z0000 bis Z3204	ZKG-Zuordnungen	0/1-kodierte Indikatorvariable, 1 falls ZKG zugeordnet
ZMK01 bis ZMK32	MKG-Zuordnungen	0/1-kodierte Indikatorvariable, 1 falls MKG zugeordnet
ZSMK01 bis ZSMK32	(sehr) schwere MKG-Zuordnungen	0/1-kodierte Indikatorvariable, 1 falls schwere oder sehr schwere ZKG innerhalb von MKG besetzt
M0230 bis M0256	Metastasen, aktiv behandelt	0/1-kodierte Indikatorvariable, 1 falls im gleichen Jahr neben Z0255 weitere ZKG aus MPG 04 besetzt ist.<br><br>27 ZKG von Z0230 bis Z0256 der MPG 04, eine differenzierbare MKG 02 basierend auf genau diesen ZKG (ZMK02, der alle Personen der MPG zugeordnet werden), 26 Pseudo-ZKG (M0230 … M0256), sofern neben der ZKG Z0255 „Metastasen, akut" im selben Jahr noch eine entsprechende weitere ZKG der MPG 04 dokumentiert ist

Tabelle A3: Anzahl Kombinationen innerhalb jeder MPG

MPG	Bezeichnung	Kombinationskennungen (Anzahl)
01	Neugeborene	ZK0101 bis ZK0103 (3)
02	Schwangerschaft, Geburt, Wochenbett	ZK0201 bis ZK0203 (3)
03	Schwerwiegende Hochkostenfälle	ZK0301 bis ZK0332 (32)
04	Aktiv behandelte bösartige Neubildungen	ZK0401 bis ZK0406 (6)
05	Mindestens eine (sehr) schwere Erkrankung	ZK0501 bis ZK0547 (47)
06	Mindestens eine moderate Erkrankung	ZK0601 bis ZK0642 (42)
07	Mindestens eine leichte Erkrankung	ZK0701 bis ZK0728 (28)
08	Mindestens eine sehr leichte Erkrankung	ZK0801 bis ZK0802(2)

Tabelle A4: Zur PBPG-Zuordnung in einzelnen MPGs genutzte ZKG sowie aus ZKGs ableitbare Merkmale

MPG PBPGs	Bezeichnung	genutzte ZKG und ggf. aus ZKG abgeleitete Merkmale
**01** P01001-P01014	Neugeborene	9 ZKG nur aus der MKG 27, differenzierbare MKG 01 bis 32 basierend auf allen ZKG (ZMK01 bis ZMK32), zwei Pseudo-ZKG, die die Dokumentation (mindestens) einer von 57 ZKG aus der MPG 03 anzeigt (ZZMK3) oder die die Dokumentation (mindestens) einer von 27 ZKG aus der MPG 04 anzeigt (ZZMK4), 3 Kombinationen, Pseudo-ZKG ZREST für Restgruppe (der alle Personen der MPG zugeordnet werden)
**02** P02001-P02012	Schwangerschaft, Geburt, Wochenbett	8 ZKG nur aus MKG 20, differenzierbare MKG 01 bis 32 basierend auf allen ZKG (ZMK01 bis ZMK32), zwei Pseudo-ZKG, die die Dokumentation (mindestens) einer von 57 ZKG aus der MPG 03 anzeigt (ZZMK3) oder die die Dokumentation (mindestens) einer von 27 ZKG aus der MPG 04 anzeigt (ZZMK4), 4 Kombinationen, Pseudo-ZKG ZREST für Restgruppe (der alle Personen der MPG zugeordnet werden)
**03** P03001-P03069	Schwerwiegende Hochkostenfälle	57 ZKG der MPG 03, 17 differenzierbare MKG basierend auf genau diesen ZKG (ZMK01 … ZMK25), 32 Kombinationen, Pseudo-ZKG ZREST für Restgruppe (der alle Personen der MPG zugeordnet werden)
**04** P04001-P04036	Aktiv behandelte bösartige Neubildungen	27 ZKG von Z0230 bis Z0256 der MPG 04, eine differenzierbare MKG 02 basierend auf genau diesen ZKG (ZMK02, der alle Personen der MPG zugeordnet werden), 12 Pseudo-ZKG (M0230 … M0256), sofern neben der ZKG Z0255 „Metastasen,

**MPG PBPGs**	**Bezeichnung**	**genutzte ZKG und ggf. aus ZKG abgeleitete Merkmale**
		aktiv behandelt“ im selben Jahr noch eine entsprechende weitere ZKG der MPG 04 dokumentiert ist, 6 Kombinationen
**05** P05001-P05106	(Sehr) Schwere Erkrankungen aus mindestens 1 MKG	170 ZKG mit Schweregrad-Einstufung „schwer“ oder „sehr schwer“, differenzierbare MKG basierend auf genau diesen ZKG (ZMK01 … ZMK31),47 Kombinationen, Pseudo-ZKG ZREST für Restgruppe (der alle Personen der MPG zugeordnet werden)
**06** P06001-P06087	Mindestens eine moderate Erkrankung	87 ZKG mit Schweregrad-Einstufung „moderat“, differenzierbare MKG basierend auf genau diesen ZKG (ZMK01 … ZMK29), 42 Kombinationen, Pseudo-ZKG ZREST für Restgruppe (der alle Personen der MPG zugeordnet werden)
**07** P07001-P07068	Mindestens eine leichte Erkrankung	86 ZKG mit Schweregrad-Einstufung „leicht“, differenzierbare MKG basierend auf genau diesen ZKG (ZMK01 … ZMK31), 28 Kombinationen, Pseudo-ZKG ZREST für Restgruppe (der alle Personen der MPG zugeordnet werden)
**08** P08001-P08033	Mindestens eine sehr leichte Erkrankung	87 ZKG mit Schweregrad-Einstufung „sehr leicht“, differenzierbare MKG basierend auf genau diesen ZKG (ZMK01 … ZMK32), zwei Kombination, Pseudo-ZKG ZREST für Restgruppe (der alle Personen der MPG zugeordnet werden)
**09** P09001	Inanspruchnahme ohne Erkrankung	(ausschließlich mit Pseudo-ZKG Z0000)
**10** P10001	Keine Inanspruchnahme	(ausschließlich mit Pseudo-ZKG Z0000)

Tabelle A5: Kennzahlen zur Diskrimination von Gesamtkosten durch unterschiedliche Gruppierungsebenen – PopGrouper 1.0 – ausschließlich Versicherte mit dokumentierten Kosten

	**mean squared error – MSE [%]**				**mean absolute error – MAE [%]**			
	**TrD**	**TeD**	**Gesamtpop.**		**TrD**	**TeD**	**Gesamtpop.**	
**Erhebungsjahr**	**2022**	**2022**	**2021**	**2023**	**2022**	**2022**	**2021**	**2023**
Anzahl Versicherte (in Tsd., roh)	*6.642*	*1.659*	*8.609*	*8.170*	*6.642*	*1.659*	*8.609*	*8.170*
MSE in Mio. €²; MAE in €²	*160,39*	*166,90*	*160,25*	*182,64*	*4.193*	*4.188*	*4.003*	*4.555*
Population insgesamt	100,0	**100,0**	100,0	100,0	100,0	**100,0**	100,0	100,0
Makro-PopGroups (MPGs)	82,0	**82,8**	82,8	81,0	72,4	**72,5**	71,1	70,7
Führende Erkrankungen (FEs)	70,1	**70,5**	71,3	70,1	62,7	**63,8**	62,6	62,7
Prä-Basis-PopGroups (PBPGs)	67,6	**68,1**	69,0	67,5	61,2	**61,3**	61,1	61,2
Basis-PopGroups (BPGs)	66,2	**66,7**	67,7	66,1	60,4	**60,5**	60,3	60,4
PopGroups (PGs)	61,6	**61,5**	62,9	61,5	58,8	**58,8**	58,5	58,8
PopGroup Kostenstrata (PGKs)	67,6	**68,2**	69,0	67,7	61,7	**61,8**	61,6	61,7

gewichtete Berechnungen zu annualisierten Gesamtkosten, nach Gruppierung verbleibende MSE- bzw. MAE-Anteile jeweils bezogen auf MSE- bzw. MAE-Werte in der nicht gruppierten Population insgesamt (hier ausschließlich Versicherte mit Zuordnung zu den MPG von 01 bis 09)

 © The Author(s).
